# Hederagenin Induces Apoptosis of Human Hepatoma HepG2 Cells *via* the Mitochondrial Pathway

**DOI:** 10.2174/0113862073254353230925074944

**Published:** 2023-10-04

**Authors:** Zhuo Liu, Xiaoning Tan, Lian Peng, Wenhui Gao, Puhua Zeng

**Affiliations:** 1 Affiliated Hospital, Hunan Academy of Traditional Chinese Medicine, Changsha 410006, P.R. China;; 2 College of Traditional Chinese Medicine, Hunan University of Chinese Medicine, Changsha, 410208, P.R. China

**Keywords:** Hederagenin, liver cancer, apoptosis, mitochondrial pathway, prognosis, pro-apoptosis

## Abstract

**Objective:**

The objective of this study is to assess the antitumor effects of hederagenin (HDG) in liver cancer (LC) cells and explore the related mechanisms.

**Materials and Methods:**

HepG2 cells were treated with HDG and cisplatin, respectively. The CCK8 assay was used to detect cell activity, DAPI staining was used to detect the proportion of living cells, TUNEL assay to detect the proportion of apoptotic cells, flow cytometry to detect the membrane potential, fluoroscopic electron microscopy to detect microstructural changes to the mitochondrial, and western blot analysis and high-content screening to detect apoptosis-related proteins.

**Results:**

Treatment with HDG inhibited the growth of HepG2 cells, decreased the proportion of viable cells, increased the proportion of apoptotic cells, and significantly increased the proportion of cells in the G1 phase. Fluorescence staining showed that HDG damaged the mitochondria of HepG2 cells and significantly decreased the number of mitochondria. Flow cytometry showed that HDG decreased the mitochondrial membrane potential of HepG2 cells. Observations by electron microscopy showed that HDG caused swelling and vacuole formation of the mitochondria of HepG2 cells. HDG significantly reduced the average fluorescence intensity of Bcl-2 in HepG2 cells and significantly increased that of the pro-apoptosis proteins Bax, Cytochrome-c, and Caspase-3.

**Conclusion:**

HDG induced apoptosis of HepG2 cells *via* the mitochondrial pathway.

## 1 INTRODUCTION

Liver cancer (LC), which is the sixth most common cancer and the second leading cause of cancer-related death worldwide, is characterized by insidious onset, rapid progression, a high malignancy rate, and poor prognosis [1]. Current treatment strategies for LC include surgery, immunotherapy, chemotherapy, and radiation therapy [2]. However, these options are limited by adverse reactions and the high recurrence rate of LC. Therefore, novel treatment strategies are urgently needed to improve patient outcomes.

Various extracts of natural herbs have been shown to enhance immunity and improve quality of life [3]. Hederagenin (HDG), (3β, 4α)-3,23-dihydroxy olean-12-ene-28-acid (molecular formula C_30_H_48_O_4_), is a pentacyclic triterpenoid compound that is widely distributed in a variety of medicinal plants, such as English ivy (Hedera helix L.), Japanese teasel (Dipsacus asper), Manchurian clematis (Clematis mandshurica), Chinese anemone (Pulsatilla chinensis), and varieties of Honeysuckle (Lonicera sp.). Recent studies have found that HDG has antitumor, antidepressant, antibacterial, anti-inflammatory, and antidiabetic effects [4]. At the molecular level, HDG is reported to induce the accumulation of reactive oxygen species (ROS) by blocking autophagic flux potentiated by the cytotoxicity of cisplatin and paclitaxel in lung cancer cells and to induce death of cisplatin-resistant head and neck cancer cells *via* inhibition of antioxidant activity [5, 6]. HDG has anticancer activity *in vivo* and *in vitro* [7]. Liu found HD-induced accumulation of ROS by blocking autophagic flux potentiated the cytotoxicity of cisplatin and paclitaxel in lung cancer cells [5]. Kim found HD induces cell death in resistant HNC cells *via* the Nrf2-ARE antioxidant pathway [6].

Mitochondria are organelles existing in liver cancer cells [8]. They maintain the homeostasis of the cell energy environment by regulating cell metabolism and reactive oxygen species [9]. Their mechanism is related to the development of triggering apoptotic factors into cascade apoptosis [10]. It can be seen that mitochondria mediated endogenous apoptosis can inhibit the proliferation of liver cancer cells.

Therefore, the aim of the present study was to investigate the effect of HDG against LC cells and elucidate the mechanism underlying the ability of HDG to induce apoptosis.

## 2 MATERIALS AND METHODS

### 2.1 Cell Source and Culture

Human hepatocellular carcinoma HepG2 cells were obtained from the Central Laboratory of the Hunan Academy of Traditional Chinese Medicine and cultured in high-glucose Dulbecco's modified Eagle's medium supplemented with 10% fetal bovine serum at a constant temperature of 37°C under an atmosphere of 5% CO_2_/95% air. Cells in the logarithmic growth phase were used for the experiments.

HepG2(SCSP-510) was derived from the Cell bank of Shanghai Chinese Academy of Sciences (Shanghai, China) Identifier: CSTR:19375.09.3101HUMSCSP510. The cells were cultured in a complete medium (DMEM; Identifier:CSTR:19375.09.3101HUMSCSP510 after academy of science (Shangai, China) Procell, Wuhan, China) containing 10%(v/v) fetal bovine serum (FBS;CELL-BOX), HepG2 cells were incubated in a constant temperature of 5% CO_2_ at 37°C.

### 2.2 Drug Preparation

HDG (5 μg) was dissolved in dimethyl sulfoxide (1 mL) and a complete medium was added to obtain a 100-μM solution. Cisplatin (20 μg) was dissolved in pure water (1 mL).

### 2.3 Chemical Reagents

HDG (HY-N0256,purity≥98%) was obtained from MedChemExpress Co.,Ltd. (USA). Cisplatin (H37021358, purity ≥98%) was obtained from Qilu Pharmaceutical Co., Ltd. (Shandong, China). CCK8 Kit (K1018) was purchased from APExBIO Company (Houston, USA). The cell cycle and apoptosis analysis kit (C1052) and enhanced mitochondrial membrane potential detection kit (JC-1)(C2003S) was obtained from Shanghai biyuntian Biotechnology Co., Ltd. (Shanghai, China), rabbit anti-BAX (E-AB-10049), Bcl-2 (E-AB-22004), Caspase-3 (E-AB-22115) were purchased Wuhan Elabscience Biotechnology Co., Ltd (Wuhan, China).

### 2.4 Cell Viability Assay

A Cell Counting Kit 8 assay kit (Abcam, Cambridge, MA, USA) was used to assess the viability of HepG2 cells treated with HDG and cisplatin. Briefly, HepG2 cells were digested with 0.25% trypsin and 150 μL of the cell suspension (~5 × 103 cells) was added to the wells of a 96-well plate. Then, HDG and cisplatin were added to the appropriate wells. After 24 h, the absorbance of each well was measured at a wavelength of 450 nm. Cell viability was assessed by comparison to untreated (control) cells.

### 2.5 Identification of Apoptotic Cells

The treated cells were cultured for 24 h at 37°C under an atmosphere of 5% CO_2_/95% air. Then, the supernatant was discarded, and 20 μL/mL of the fluorescent stain 4’,6-diamidino-2-phenylindole (DAPI) was added to each well. After staining for 10 min, the cells were washed with phosphate-buffered saline (PBS) and observed under a microscope.

### 2.6 Detection of Apoptotic HepG2 Cells

The terminal deoxynucleotidyl transferase dUTP nick end labeling (TUNEL) assay was used to detect apoptotic HepG2 cells. Briefly, treated cells were fixed with 4% paraformaldehyde for 30 min, washed twice with PBS, treated with Triton X-100 for 5 min, and washed twice with PBS. The test solution was prepared as described in the manufacturer’s instructions. The prepared cells (50 μL) were incubated at 37 °C in the absence of light for 60 min. Afterward, the staining solution was discarded and the cells were washed three times with PBS and then imaged under a fluorescence microscope [11].

### 2.7 Screening of Apoptosis-related Proteins

HepG2 cells in the logarithmic growth stage were added to the wells of black 96-well plates with the corresponding drugs for 24 h, then washed three times with cold PBS for 2 min, fixed with 4% paraformaldehyde for 30 min, and washed again three times with PBS. Afterward, the cells were treated with 0.25% triton-100 for 15 min, then washed three times with cold PBS, blocked with 5% bovine serum albumin for 30 min, and washed twice with PBS. Next, the cells were incubated overnight at 4°C in the dark with rabbit anti-mouse antibodies (dilution, 1:100) against Caspase-3, Cytochrome-c, Bal-2, and Bax; then washed three times with cold PBS and incubated at 37°C in the dark for 30 min with fluorescein isothiocyanate-labeled goat anti-rabbit and R-phycoerythrin-labeled goat anti-mouse secondary antibodies (dilution, 1:200). After washing three times with cold PBS, diluted DAPI was added to each well, and the plate was incubated at room temperature in the dark for 20 min. Afterward, 100 μL of PBS was added to each well. Fluorescence was detected and images were captured with a high content analysis (HCS).

### **2.**8. Cell Cycle and Apoptosis Detection by Flow Cytometry

After 24 h of drug intervention, the cells were collected, digested, centrifuged at 1000 × *g* for 5 min, and washed two times with PBS. Afterward, 10 µL of Annexin V-fluorescein isothiocyanate (20 µg/mL) and 400 μL of buffer were added to each well, and the plate was incubated at 4°C in the dark for 15 min and then at room temperature for 10 min. Finally, the proportion of apoptotic cells was determined by flow cytometry.

### 2.9 Detection of Mitochondrial Membrane Potential of HepG2 Cells

After washing two times with PBS, the treated cells were incubated with 1 mL of JC-1 dye for 20 min at 37°C, then washed twice with JC-1 buffer, mixed with culture medium, and imaged under a fluorescence microscope.

### 2.10 Detection of Mitochondrial Membrane Potential by Flow Cytometry

The treated cells (1 × 106) were digested, washed with cold PBS, centrifuged at for 3-4 min at 600 × *g* and 4°C, suspended in JC-1 dye working solution, incubated for 20 min at 37°C, then centrifuged for 3 min 4°C at 600 × *g* and 4°C, resuspended in JC-1 buffer, precipitated, and resuspended again in JC-1 buffer. Finally, the mitochondrial membrane potential of the cells was detected by flow cytometry.

### 2.11 Detection of Changes to the Mitochondria of HepG2 Cells by Transmission Electron Microscopy (TEM)

After 24 h of drug intervention, the cells were fixed with 2.5% glutaraldehyde solution at room temperature for about 5 min, resuspended in TEM fixing solution for 30 min at 4°C, dehydrated, and imaged by TEM.

### 2.12 Western Blot Analysis

The treated and untreated (control) HepG2 cells were lysed with radioimmunoprecipitation assay buffer on ice for 30 min, centrifuged, and the supernatant was collected. The total protein content was quantified with a bicinchoninic acid assay. Then, 50 μg aliquots of the proteins were loaded into the wells of polyacrylamide gels, separated by electrophoresis, and transferred to polyvinylidene fluoride membranes, which were blocked with 5% skimmed milk, probed with antibodies (dilution, 1:1000) against Bax, Bcl-2, cytochrome-c, and caspase-3 overnight at 4°C with shaking, washed with Tris-buffered saline and Polysorbate 20, and then incubated with secondary antibodies at room temperature for 1 h. The protein bands were visualized with an electrochemiluminescence assay kit and quantified with ImageJ software (https://imagej.nih.gov/). The experiment was repeated three times.

### 2.13 Statistical Analysis

All statistical analyses were performed with GraphPad Prism 7 software (GraphPad Software, Inc., San Diego, CA, USA). The data of multiple groups were compared by analysis of variance. The data are expressed as the mean ± standard deviation. A probability *p* value < 0.05 was considered statistically significant.

## 3 RESULTS

### 3.1 Inhibitory Effects of HDG Against HepG2 Cells

**Fig. (1):**
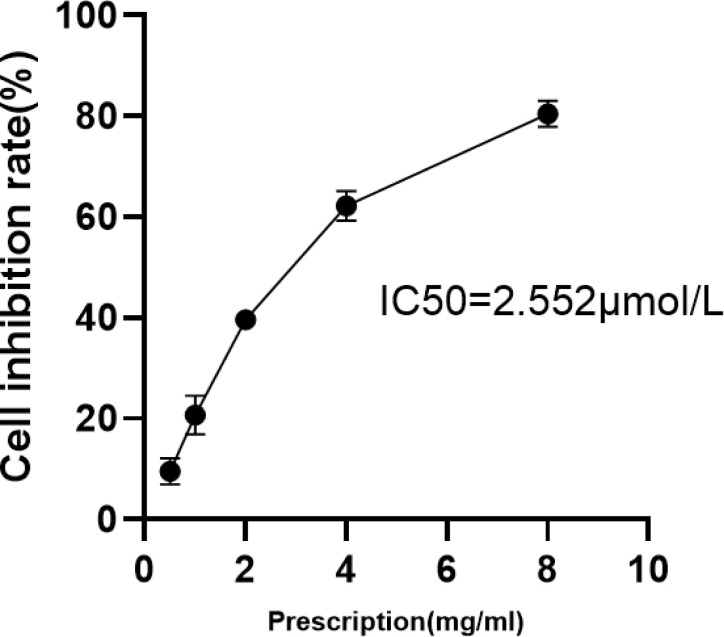
The inhibitory effect of HDG against HepG2 cells after treatment for 24 h(x̅±s, n = 3). The samples were tested in triplicate and the results are presented as the mean ± standard deviation.

The CCK8 assay results showed that the inhibitory effect of HDG against HepG2 cells was highly dependent on the concentration. There were significant differences in the half maximal inhibitory concentrations (IC_50_) of HDG (Fig. **1**).

**Fig. (2):**
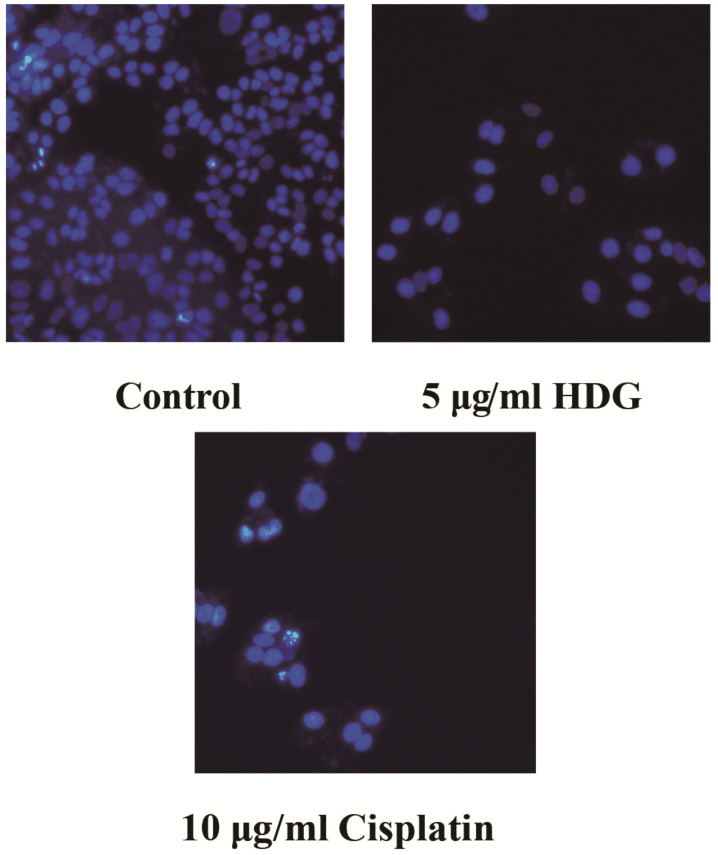
DAPI staining of treated and untreated (control) HepG2 cells (magnification, ×200).

DAPI staining was used to identify morphological changes. The number of HepG2 cells observed in HDG treatment groups was markedly decreased compared with the control group. Apoptotic cells exhibit typical changes, including shrinkage, chromatin condensation and karyorrhexis. The DAPI staining results show that HDG inhibited proliferation of HepG2 (Fig. **2**).

### 3.2 HDG Induced Apoptosis of HepG2 Cells

**Fig. (3):**
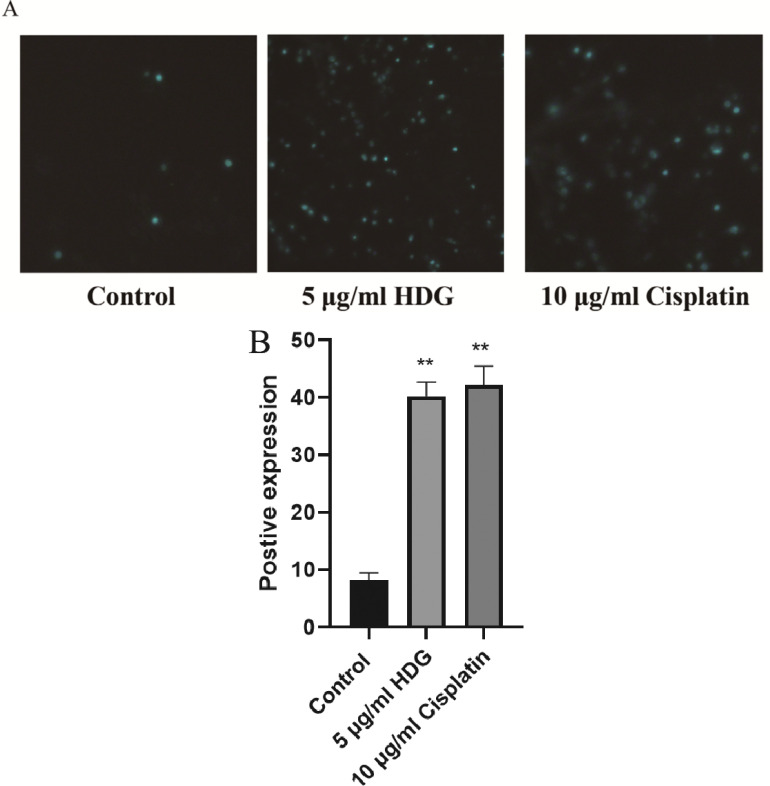
Results of the TUNEL assay. After treatment with HDG and cisplatin for 24 h, the proportions of apoptotic HepG2 cells were evaluated using the TUNEL assay. (**A**) The HepG2 cells were observed under a fluorescence microscope (magnification, ×200). (**B**) Positive expression with the TUNEL assay(Relevant data of each group (n = 6; *p < 0.05, ** *p* < 0.01 *vs.* the control group).

The results of the TUNEL assay showed that the proportion of apoptotic HepG2 cells was significantly greater in the HDG and cisplatin groups than in the control group (Fig. **3**).

### 3.3 Effect of HDG on Proliferation of HepG2 Cells

**Fig. (4):**
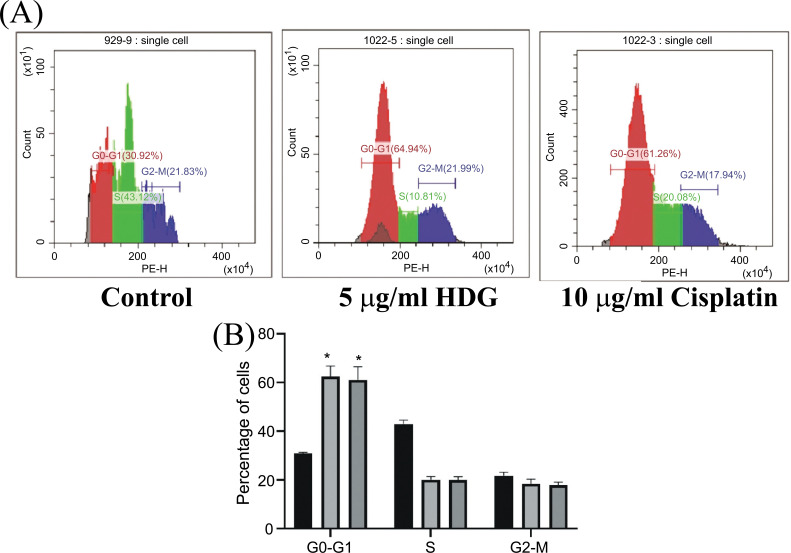
Results of cell cycle analysis. (**A**) The proportions of treated and untreated (control) HepG2 cells in various stages of the cell cycle were determined by flow cytometry, (**B**): Proportions of apoptotic HepG2 cells by flow cytometry (Relevant data of each group (n = 6; *p < 0.05, ** *p* < 0.01 *vs.* the control group).

**Fig. (5):**
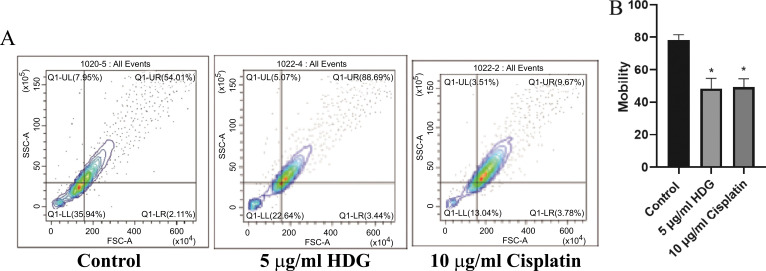
Detection of apoptotic HepG2 cells. (**A**) The proportions of treated and untreated (control) apoptotic HepG2 cells were determined by flow cytometry. (**B**) Stage of HepG2.

The proportions of HepG2 cells in the G0/G1 phase were significantly greater in the HDG and cisplatin groups than in the control group. The proportions of HepG2 cells in the S and G2 phases were decreased in the HDG and cisplatin groups as compared with the control group, but the differences were not statistically significant (Fig. **4**). The results of the apoptosis detection by flow cytometry showed that the proportion of apoptotic HepG2 cells was significantly greater in the HDG and cisplatin groups than in the control group (Fig. **5**).

### 3.4 Effect of HDG on Mitochondrial Membrane Potential of HepG2 Cells

The mitochondrial membrane potential of HepG2 cells was significantly decreased in the HDG and cisplatin groups as compared to the control group.

**Fig. (6):**
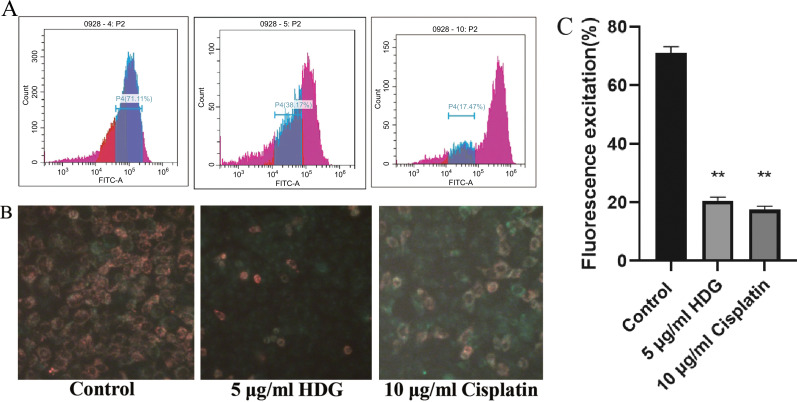
Mitochondrial membrane potential. (**A**) The mitochondrial membrane potential of HepG2 cells was assessed by flow cytometry. (**B**) Images of HepG2 cells depicting differences in mitochondrial membrane potential. Treated and untreated (control) HepG2 cells were imaged under a fluorescence microscope to assess mitochondrial membrane potential (magnification, ×200). (**C**) Fluorescence excitation (%) of mitochondrial membrane potential(Relevant data of each group (n = 6; *p < 0.05, ** *p* < 0.01 *vs.* the control group).

In addition to the decrease in the mitochondrial membrane potential of HepG2 cells in the HDG and cisplatin groups, cell adhesion was weakened (Fig. **6**).

### 3.5 HDG Damaged the Mitochondria of HepG2 Cells

**Fig. (7):**
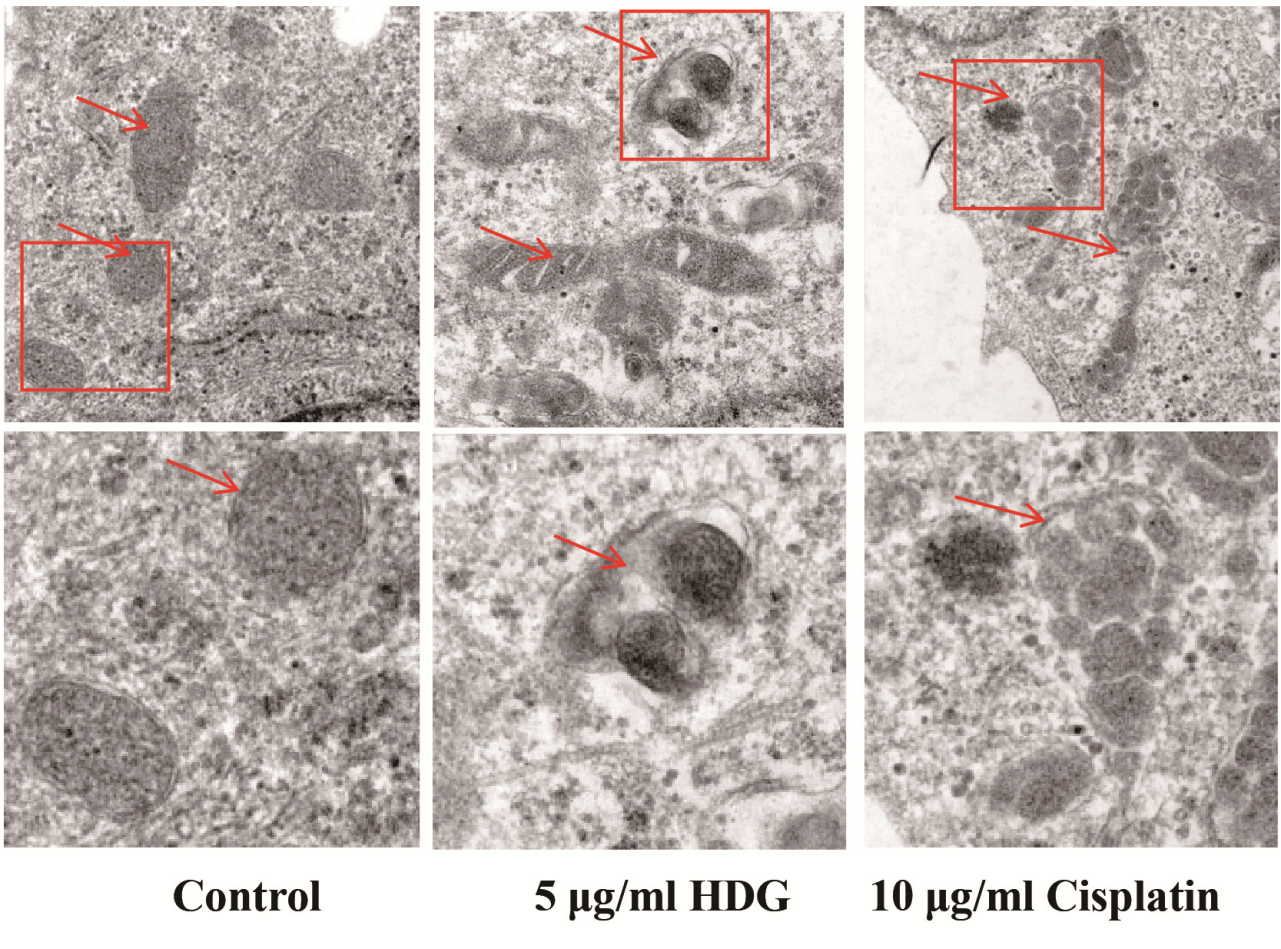
Morphological changes to mitochondria observed by TEM. Morphological changes to mitochondria of HepG2 cells were observed by TEM.

HDG damaged the mitochondria, thereby triggering apoptosis of HepG2 cells. As compared with the control group, the mitochondria of the HDG and cisplatin groups were swollen with the formation of vacuoles, disordered ridge-like structures, and obvious pyknosis (Fig. **7**).

### 3.6 Effects of HDG on the Protein Levels of Bax, Bcl-2, Cytochrome-c, and Caspase-3

**Fig. (8):**
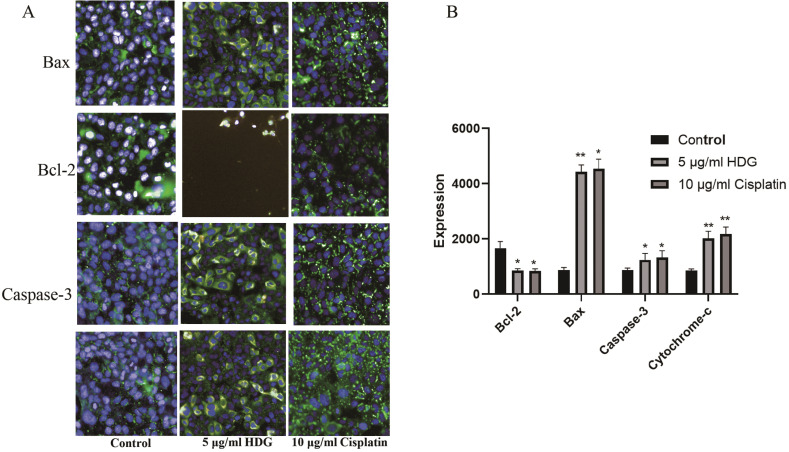
Protein expression levels of Bax, Bcl-2, Caspase-3, and Cytochrome-c. (**A**) HepG2 cells were treated with HSC and the protein expression profiles of Bax, Bcl-2, Caspase-3, and Cytochrome-c were observed. (**B**) Protein expression profiles of HSC cells (Relevant data of each group (n = 6; *p < 0.05, ** *p* < 0.01 *vs.* the control group).

**Fig. (9):**
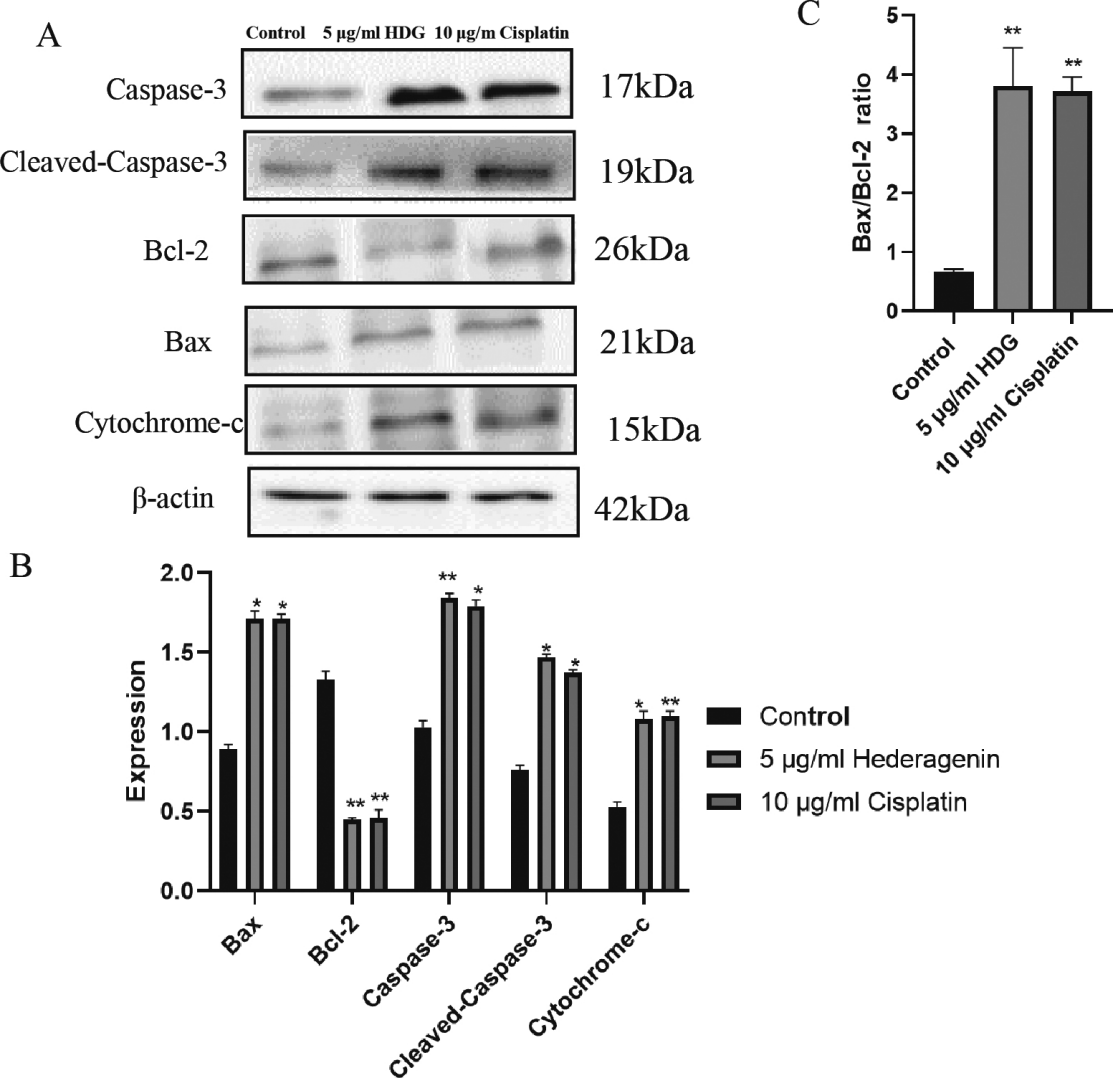
Western blot analysis of Bax, Bcl-2, Caspase-3, Cleaved-Caspase-3and Cytochrome-c. (**A**) Protein expression levels of Bax,Bcl-2,Caspase-3,Cleaved-Caspase-3 and Cytochrome-c of treated and untreated (control) HepG2 cells were determined by western blot analysis. (**B**) Protein expression levels determined by western blot analysis (Relevant data of each group (n = 6; *p < 0.05, ** *p* < 0.01 *vs.* the control group). (**C**) The quantification of the Bax/Bcl-2 ratio by western blot analysis. (Relevant data of each group (n = 6; *p < 0.05, ** *p* < 0.01 *vs.* the control group).

As compared with the control group, the protein expression levels of Cytochrome-c, Bax, Cleaved-Caspase-3 and Caspase-3 were significantly increased in the HDG and cisplatin groups, while protein expression of Bcl-2 was significantly decreased (Figs. **8** and **9**). The quantification of the Bax/Bcl-2 ratio of HDG and cisplatin groups was higher than the control group (Fig. **9C**).

## 4 DISCUSSION

HDG is abundant in nature and widely distributed in various plants. Pharmacological studies of HDG have mainly focused on the antitumor, antidepressant, antibacterial and anti-inflammatory activities of HDG. However, the underlying mechanisms remain unclear. Previous studies have reported that HDG significantly inhibited the growth of HepG2 cells and that the antitumor effect may be closely related to apoptosis initiated *via* the mitochondrial pathway, although the detailed mechanism has not been reported [12].

Apoptosis can effectively inhibit the growth and proliferation of tumor cells, thus inhibiting the occurrence and development of tumors [13]. Inducing tumor cell apoptosis can kill cancer cells while effectively avoiding damage to normal cells [14]. Therefore, it is an important means to effectively inhibit the proliferation of cancer cells [15]. Apoptosis is often manifested by the reduction of cell volume, changes in organelles and the appearance of apoptotic bodies. Mitochondria, death receptors and endoplasmic reticulum are the three mechanisms of apoptosis, which induce programmed death pathways through apoptotic signals, thus enabling effective treatment of tumor diseases [16].

In recent years, apoptosis initiated *via* the mitochondrial pathway has become a hot research topic for the prevention and treatment of LC [17]. The dysfunctional mitochondria of LC cells are selectively phagocytized by phagosomes to maintain mitochondrial homeostasis and energy production [18]. In addition, mitochondria can inhibit the proliferation and migration of hepatoma cells by triggering apoptosis [16]. Permeabilization of the outer membrane of the mitochondria releases Cytochrome-c, leading to activation of Bax-related pathways that induce apoptosis [19, 20].

The results of *in vitro* studies have shown that HDG inhibits the proliferation and metastasis of HepG2 cells by initiating apoptosis *via* the mitochondrial pathway. The pro-apoptosis activities of HDG involve reductions in mitochondrial membrane potential. During the apoptosis process, vacuoles form in the mitochondria, accompanied by the appearance of disordered ridge-like structures. The results of the present study demonstrated that HDG significantly decreased the average fluorescence intensity of Bcl-2 in HepG2 cells and significantly increased that of the pro-apoptosis proteins Bax, Cytochrome-c, and Caspase-3. In addition, HDG disrupts energy production by the mitochondria of LC cells.

## CONCLUSION

This study provides important information regarding the therapeutic effects of HDG against LC. However, the mechanisms underlying the antitumor effects of HDG remain unclear. Therefore, further studies are warranted to elucidate the mechanism employed by HDG to inhibit the proliferation of LC cells.

## LIMITATION

An apparent limitation of the method is it's an animal experiment, not a clinical sample. It can be further verified by clinical trials.

## Data Availability

The data used to support the findings of this study are available from the corresponding author [P.Z.] upon request.

## LIST OF ABBREVIATIONS

DAPI: Diamidino-2-phenylindole
HCS: High Content Analysis
HDG: Hederagenin
IC: Inhibitory Concentrations
LC: Liver Cancer
PBS: Phosphate-Buffered Saline
ROS: Reactive Oxygen Species
TEM: Transmission Electron Microscopy
TUNEL: Transferase dUTP Nick End Labeling
